# Incidence and factors associated with failed spinal anaesthesia among patients undergoing surgery: a multi- center prospective observational study

**DOI:** 10.1186/s12871-024-02484-y

**Published:** 2024-04-05

**Authors:** Atalay Eshetie Demilie, Zewditu Abdissa Denu, Yosef Belay Bizuneh, Endale Gebreegziabher Gebremedhn

**Affiliations:** 1 Department of Anaesthesia, College of Medicine and Health Sciences, Bahir Dar University, Bahir Dar, Ethiopia; 2 Department of Anaesthesia, School of Medicine, Gondar College of Medicine and Health Sciences, University of Gondar, Gondar, Ethiopia

**Keywords:** Spinal anaesthesia, Failure, Local anaesthetics, Factors

## Abstract

**Background:**

Failed spinal anaesthesia causes prolonging of operation time, insufficient analgesia for surgery and needs repeating spinal anaesthesia which in turn causes local anaesthesia toxicity, high spinal and total spinal, and conversion to general anaesthesia. However, the problem remains unexplored in Amhara regional state comprehensive specialized hospitals.

**Objective:**

To determine incidence and factors associated with failed spinal anaesthesia among patients undergoing surgery in selected Amhara National Regional State comprehensive specialized hospitals, Northwest Ethiopia, 2023.

**Methods:**

Multi-center prospective observational study was conducted. Data was collected using questionnaire and checklist. All consecutive scheduled emergency and elective patients were included in the study. Data was transformed from Epi data to SPSS and logistic regression analysis was done. Both crude and adjusted odds ratio were used to assess the strength of association. Variables with a *p*-value of less than 0.05 were considered as statistically significant.

**Results:**

A total of 532 patients were included in this study with a response rate of 98%. Incidence of failed spinal anaesthesia was 22.4% (CI = 19-25.9). Emergency surgery (AOR = 7.01, CI = 4.5–12.7), dose of bupivacaine of ≤ 10 mg (AOR = 3.02, CI = 1.3–10.2), work experience of anaesthetist < 2 years (AOR = 3.1, CI = 1.7–5.72), bloody CSF (AOR = 8.5, CI = 2.53–18.5), hyperbaric local anaesthetic drug (AOR = 3.3, 95% CI = 3.2–8.2) and local anaesthetist without adjuvants (AOR = 5.25, CI = 2.62–14.2) were associated failed spinal anaesthesia.

**Conclusion and recommendation:**

The incidence of failed spinal anaesthesia was high in Amhara Region comprehensive specialized hospitals. We suggest that anaesthesia providers should minimize failure by using adjuvants and appropriate dose of local anaesthetic. Additionally, simulation training should be given for anaesthesia trainees to improve their skills and to produce competent professionals.

## Background

Spinal anaesthesia is a type of regional block that involves the temporary numbness of sensory, motor, and sympathetic nerves which is achieved by injecting a local anaesthetic and other agents into the subarachnoid space, which surrounds the spinal cord [1]. The mechanism of action for spinal anesthesia is the distribution of the local anaesthetic through the subarachnoid fluid, which bathes the nerve roots and results in the desired blockage [2].

Spinal anesthesia is the preferred choice for lower abdomen and lower extremity surgeries such as orthopedic, urologic, gynecologic, general surgery, and caesarian section due to its rapid onset, predictability, and reliable blockage [3]. It also provides excellent postoperative pain relief without the risks associated with general anaesthesia, such as pulmonary aspiration [4, 5].

It is a preferred choice of anesthesia over general anesthesia because of its simplicity, minimal drug use, reduced intraoperative blood loss, maintenance of cardiac and pulmonary function, prevention of pulmonary aspiration, and avoiding airway complications [6, 7]. When compared to general anesthesia, spinal anesthesia reduces cardiopulmonary complications and the 30-day mortality rate [6]. Patients and anesthesiologists often prefer spinal anesthesia to general anesthesia due to its advantages of providing both anesthesia and analgesia simultaneously, preventing serious respiratory complications associated with general anesthesia (GA), and high patient satisfaction [7].

The success of spinal anaesthesia may depend on the practitioner’s training, the characteristics of the patient population, or the patient’s position during the injection. The most common causes for failure to access the subdural space are reported to be an inappropriate patient posture, the dosage of the drug used, baricity, the patient’s position during surgery, and the length of the procedure [8].

Even though administration of spinal anaesthesia is relatively straightforward the possibility of failure has long been recognized in early of quote being taken from the work of Gaston Labat, the ‘father’ of modern regional anaesthesia [9]. Failed spinal anaesthesia can be defined as complete, partial or incomplete spinal block within 15–20 min after intrathecal injection of local anesthetics into subarachnoid space and requiring supplemental analgesia or conversion to general anaesthesia due to different reasons like inadequate volume of LA, anatomical variations and skill of anesthesia providers [10–12]. Failed spinal anaesthesia might be a source of pain, anxiety, and psychological trauma to the patient and a concern and even a sequelae for medico- legal to anaesthesia providers [13, 14].

Recently, spinal anaesthesia failure after injection of local anaesthetic to subarachnoid pace has been reported most frequently in various literatures [15–18]. However, any procedure which is converted from spinal anaesthesia to general anaesthesia or required analgesia and sedation due to an unexpectedly long surgery (> 2 h) is not considered as failed spinal anaesthesia [19].

Failed spinal anaesthesia has several drawbacks, including prolonged operating times, insufficient analgesia that is insufficient for surgery, repeated procedures after a failed dural puncture, which can cause local anaesthesia toxicity, high spinal and total spinal, and conversion to general anaesthesia, which puts patients at higher risk for complications related to general anaesthesia and raises respiratory risks [17, 20].

Due to the nature of the trauma, spinal anesthesia fails frequently with orthopedic patients because pain affects patient’s position and may necessitate multiple dural puncture attempts [16]. In addition, an increase in the number of attempts is recorded as a reason for failure when spinal anesthesia is administered in the lateral position for procedures related to the hip fracture [16].

In the event that a spinal anesthesia fails, clinicians have several alternative solutions [14]. They may attempt to repeat the spinal anesthesia, provide pain relief through opioids or sedation, use local anesthesia at the site of the operation, or switch to general anesthesia [14]. However, these alternatives may result in respiratory problems that increase the risk of complications and may require postponing the surgery [21].

The incidence of failed spinal anesthesia has been studied in a number of countries and most of studies show that the failure rate of spinal anesthesia ranges from 1 to 17% [1, 22, 23]. The difference might be due to variation in clinical settings, population variation, and experience of anaesthesia provider [24].

Age, sex, height, body mass index (BMI), surgical site ,vertebral interspace used, needle gauge and type, number of attempts, type of local anesthetics (baricity) and dose of LA, use of adjuvants, presence of paresthesia during procedure, presence of blood on aspiration of CSF, and patient position during procedure are some of the factors that contribute to failed spinal anaesthesia [16].

Failure of a spinal anaesthetic is an event of significant concern for both the patient and anaesthetist even when it is immediately apparent, but it can have serious consequences (clinical and medico-legal) if the problem only becomes evident once surgery has started [14]. To decrease the failure the trainee anesthetists should avoid over-selling the technique, especially in the early days of unsupervised practice [14].

Options for managing an inadequate block or failed spinal anaesthesia include repeating the injection of local anesthetics, supplementation with local anaesthetic infiltration by the surgeon, use of systemic sedation or analgesic drugs, converted to general anaesthesia and depend on the urgency of surgery, airway and cardiopulmonary risks of the patient may defer the surgery and prepare other options [14].

Localization of the subarachnoid space is sometimes challenging, and the absence of a free flow of CSF through the needle increases the risk of failure [24]. With advance in technology and medical care, life expectancy and the number of geriatric patients are increasing and these patient groups have concomitant medical problem like different genitourinary problems with limited physiologic adaptation and SA given frequently is an ideal anesthetic technique for these patient groups, the advantage is hampered and sometimes things may get complicated with failure of spinal anaesthesia [25].

In Ethiopia comprehensive studies on incidence and risk factors encompassing all surgical procedures have not yet been conducted. Even though some studies conducted only among obstetric mother and difficult to generalize about general populations due to many factors affecting failed spinal anaesthesia. Hence, this study aimed to determine the incidence and factors associated with failed spinal anaesthesia among patients undergoing surgery under spinal anaesthesia in selected Amhara National Regional State comprehensive specialized hospitals, Northwest Ethiopia, 2023.

## Materials and methods

### Study design and study period:

multi-center prospective observational study was conducted from April 10 to June 10, 2023 in selected Amhara National Regional State comprehensive and specialized referral hospitals, Northwest Ethiopia.

### Study area

#### The study was conducted in three selected hospitals namely:

 the University of Gondar comprehensive specialized hospital, Felege hiwot comprehensive specialized hospital and Tibebe Ghion comprehensive specialized hospital which are located in Amhara Region Northwest Ethiopia.

#### University Gondar comprehensive specialized hospital:

Is located in central Gondar administrative zone, Amara National Regional state, which is far from about 750 km Northwest of Addis Ababa (the capital city of Ethiopia). Currently the University of Gondar comprehensive specialized hospital has 14 functional operation rooms: one ophthalmic surgery, two fistulas, two obstetric, seven general operation room, one orthopedic and one day case surgery .On average around 270 patients are operated under spinal anaesthesia per month according to logbook report of the different departments.

#### Felege hiwot referral comprehensive hospital:

Found at Bahir Dar city which has two orthopedic operation rooms, four general surgery operation room and two gynecologic and obstetrics operation room and on average 245 cases are operated under spinal anaesthesia per month according to hospital reports.

#### Tibebe Ghion comprehensive specialized hospital

Found at Bahir Dar city which has seven general operation rooms, two obstetric operation room and two orthopedic operation rooms on average and on average around 260 patients are being operated under spinal anaesthesia per month according to hospital reports.

#### Source population:

All patients who underwent surgery under spinal anaesthesia in Amhara National Regional State comprehensive specialized hospitals.

#### Study population:

All patients who underwent surgery under spinal anaesthesia during study period.

#### Inclusion criteria:

All adult patients who underwent surgery under spinal anaesthesia.

#### Exclusion criteria:

Patients on spinal anaesthesia with peripheral nerve block like lumbar plexuses, sciatic and femoral nerve blocks on preoperative period, patients on combined spinal epidural anaesthesia, patients take analgesia and sedation or repeat SA or convert to general anaesthesia before 20 min of intrathecal drug administration.

#### Sample size:

To determine the sample size single population proportion formula was used. Since there is no previous study done in a similar setting on general surgical population we take proportion of 50% by assuming 95% of confidence interval with 5% margin of error, and finally the sample size for the study is calculated as.

$$\begin{array}{l}n = \frac{{{{\left( {Z{\raise0.7ex\hbox{$\alpha $} \!\mathord{\left/{\vphantom {\alpha 2}}\right.\kern-\nulldelimiterspace}\!\lower0.7ex\hbox{$2$}}} \right)}^2}\rho \left( {1 - \rho } \right)}}{{{\epsilon ^2}}}\\n = \frac{{{{\left( {1.96} \right)}^2} * 0.5\left( {1 - 0.5} \right)}}{{{{(0.05)}^2}}}\end{array}$$

  *n* = 384.16, N ∼ **385**.

**Fig. 1:**
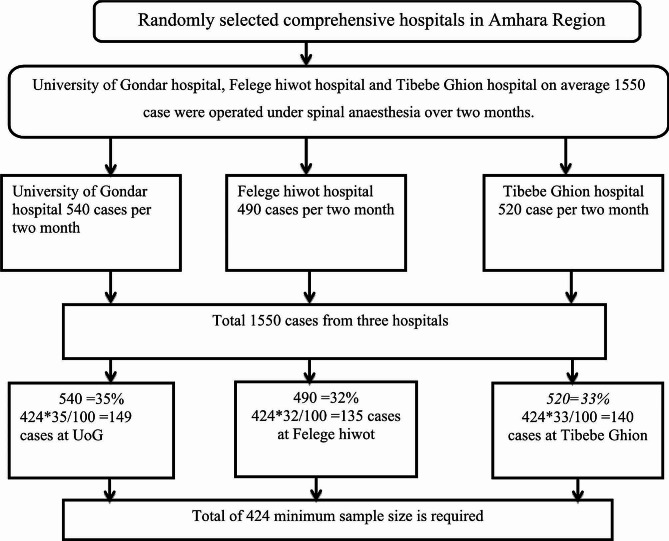
Sample size allocation for each hospital

By considering 10% of non-response rate the total sample size 424. By using proportion allocation formula depend on two month available data which was 1550 cases done under spinal anaesthesia on three hospitals (Fig. 1).

### Sampling technique

All consecutive surgical patients who were operated under spinal anaesthesia in three selected comprehensive and specialized hospitals during the study period were included.

#### Dependent variable:

Failed spinal anaesthesia (Yes, No).

### Independent variables

#### Patient factors:

Sex, age, height, weight, ASA status, anatomic deformity, palpability of spinous process (grade) and body mass index.

#### Anaesthetist factors:

experience, level of education, number of attempts.

#### Drug factors:

drug (bupivacaine, lidocaine), dose, volume, baricity (hyperbaric, isobaric, hypobaric), adjuvants (dose and type like pethidine, morphine, fentanyl).

#### Surgery factors:

patient position during surgery, duration of surgery and, type of surgery.

#### Technique related factors:

site of needle insertion, vertebral needle approach (midline, paramedian), needle type and size, patient position (sitting, lateral & prone), speed of injection, not reached to the subarachnoid space and position after spinal anaesthesia.

#### Spinal block status:

CSF flow seen, no CSF flow, CSF color (dry tape, clear, bloody), block (partial, complete), time taken to achieve complete block, management of FSA (spinal anaesthesia repeated, converted to GA).

### Operational definitions:

#### Adequate block:

Condition where the planned surgery can be conducted after sensory and motor block checked without pain and discomfort [26].

#### Modified Holmen sensory blockage scale:

Grade 1 = full sensation, Grade 2 = weak sensation, Grade 3 = Recognized as light touch, Grade 4 = Loss of sensation [27].

#### Bromage scale:

an accepted tool to assess the intensity of motor block of lower extremity write the scales; where 0 = unable to move feet or knees, 1 = able to move feet only, 2 = just able to move knees and 3 = full flexion of knee and feet [28].

#### Failed spinal anesthesia:

Failed spinal anesthesia can be defined as partial or incomplete spinal block within 15–20 min after injection and requiring supplemental analgesia or conversion to general anaesthesia [10].

#### Spine palpability:

grade 1and 2; where the spinous process can be touched [29].

#### Poorly palpable spine:

grade 3and 4; where the spinous process can felt but with difficulty [30].

Where Grade 1 (spinous processes are visible), Grade 2 (spinous processes are not seen but easily palpated), Grade 3 (spinous processes are not seen but barely palpable under the thumb) and Grade 4 (none of the previous) [29, 30].

#### Position:

Refers to patient position during performing the spinal anesthesia, sitting, lateral or prone.

### Data collection tool and procedure

Data collection was done using structured and pretested questionnaire and checklist. The structured questionnaire was filed by the data collector, using relevant information from the patient chart demographic data (sex, age, height, weight, BMI, ASA Status) and direct observation (needle gauge, number of attempts, CSF flow, normal or deformed, patient position during the procedure) and by asking anesthetist (educational level and work experience of anesthetist) of the procedure in the operation room.

### Data quality assurance

Data were collected by six trained data collectors guided by three supervisors. Training and orientation were given to data collectors and supervisors for one day by the principal and co investigators on the aim and objective of the study, how to approach study participants, how to use the questionnaire, on how to supervise and collect the data. During data collection, all data were collected and properly filled on the prepared format and supervisors supervised the data collector and checked for completeness every day.

### Data processing and analysis procedure

After we collected data, the questionnaire paper was checked manually for completeness and then it was coded at Epi data version 4.6 and was transformed from Epi data to SPSS and analyzed using SPSS Version 22. Descriptive statistics were carried out and the results were presented using text, tables, and graphs. Assumption of binary logistic regression was checked including chi-square test, multi collinearity was checked and continuous variables were normally distributed presented using mean and standard division. Hosmer –Lemeshow test was used to check the fitness of the model. Logistic regression analysis was used to determine the independent variables which have association with the dependent variable. Both crude and adjusted odds ratio were used to assess the strength of association between dependent and independent variables. Those variables with *p* value less than 0.2 from the bivariable regression analysis were fitted for the multivariable logistic regression analysis. At 95% confidence interval a *p*-value of less than 0.05 was considered as statistically significant.

### Ethical consideration

Ethical clearance was obtained from the University of Gondar, College of Medicine and Health Science ethical review committee with ethical registration number of (Ref No SOM 516;04/04/2023). Permission letter was also obtained from other hospitals .Oral informed consent was obtained from each study subject after a clear explanation during the preoperative period about the objective, purpose and as they have the right to refuse to participate in the study. Confidentiality was ensured by avoiding personal identifiers and locking the questionnaire and checklist.

## Result

### Socio-demographic characteristics of the study participants

A total of 543 patients who underwent spinal anesthesia were included in the study. However, 11 patients were excluded due to incomplete data. Therefore, the final sample size for this study was 532 patients, resulting in a response rate of 98%. Of 532 patients, 203 were operated at the University of Gondar specialized hospital (38%), 193 at Tibebe Ghion specialized hospital (36%), and 136 at Felege hiwot specialized hospital (26%) respectively (Table 1). Among the participants, 281 (53%) were females and 251 (47%) were males. The average age of the study participants was 39.05 ± 17.7, with the majority (63%) were under 40 years old (Table 1).

**Table 1:** Socio demographic characteristics of the study participants (*N* = 532)

Variables		Category			Frequency (n)	Percentage (%)
Gender		Female			251	47%
		Male			281	53%
Age	Minimum		Maximum	Range	Mean	St. deviation
	10		88	78	39.1	17.7
Height		< 1.6			127	24%
		> 1.6			405	76%
BMI		< 18.5			10	2%
		18.5–24.9			373	70%
		25-29.9			130	24%
		> 30			19	4%
Weight of the patient in kilogram		< 50			40	8%
		51–90			489 384	92%
		> 90			3	1%
Hospitals where operation done		UoGCSH			203	38%
		FHCSH			193	36%
		TGCSH			136	26%

### Factors related with patients

Of 532 study participants, 327 (61%) were ASA II, 185 (35%) ASA I and 20 (4%) ASA III respectively (Table 2). Most patients (*n* = 333, 63%) had not previous history of spinal anaesthesia, whereas 199(37%) had pervious exposure for spinal anesthesia .Additionally, most patients (*n* = 517, 97%) had not anatomic deformity along the spinous proses but fifteen patients (3%) had anatomical deformity. The spinous process was easily palpable in 79% of patients but it was poorly palpable in 21% of patients and was not palpable at all in 0.4% of patients (Table 2).

**Table 2:** ; Factors related with patients (*N* = 532)

Variables	Category		Frequency (n)	Percentage (%)
ASA Classification	ASA I		185	35%
	ASA II		327	62%
	ASA III		20	4%
Previous history of SA	Yes		199	37%
	No		333	63%
anatomic deformity	Yes		15	3%
	No		517	97%
palpability of spinous	Yes		421	79%
	No	Poorly palpable	109	21%
		Not palpable	2	0.4%
	Total		532	100%

### Technical related factors

Spinal anaesthesia was administered on sitting position for 520 (98%) patients, and on the lateral position for 12 (2%) patients respectively (Table 3). Lumbar puncture was done between L3-L4 for 475 (89%) patients. The most common vertebral approach was the median approach (91%). During lumbar puncture, most of the patients (80%) did not feel paresthesia. Most anaesthetists administered LA slowly (97%). Most anaesthetists used 24G (57%) and which were quincke type spinal needle. After administration of LA, most anesthetists put the patients on head up position (60%). The maximum number of attempts for lumbar puncture was 4 times and minimum attempts were 1 times with mean of 1.61 ± 0.742 (Table 3).

**Table 3:** Techniques of lumbar puncture (*N* = 532)

Variables	Category		Frequency (n)	Percentage (%)	
Patient position during lumbar puncture	Sitting		520	98%	
	Lateral		12	2%	
Site of lumbar puncture	L2-L3		11	2%	
	L3-L4		475	89%	
	L4-L5		46	9%	
Vertebral approach during lumbar puncture	Median		482	91%	
	Para median		35	7%	
	Taylors		15	3%	
Paresthesia during puncture	Yes		105	20%	
	No		427	80%	
Speed of injection	Fast		14	3%	
	Slow		518	97%	
Needle size in gauge	22		8	2%	
	23		219	41%	
	24		305	57%	
Immediately change of position after spinal anaesthesia	Yes	Head up	308	440	83%
		Head down	36		
		Left lateral	94		
		Right lateral	2		
	No		92	17%	
Number of spinal attempt	1–2 times		463	87%	
	3–4 times		69	13%	
Needle type	Quincke		532	100%	

### Factors related with the anaesthetist

The majority of spinal anaesthesia was administered by BSc anaesthetists (*n* = 201, 38%) and MSc students (*n* = 122, 23%) respectively (Table 4). One hundred fourteen *n* = 114 (21%) of spinal anaesthesia were administered by MSc anaesthetists. The majority of patients (81%) were anaesthetized by anaesthetists with work experience of two year and above and 19% of patients were anaesthetized by anaesthetists with work experience of less than two years (Table 4) .

**Table 4:** Educational statues of the anaesthetists (*N* = 532)

Variables	Category	Frequency (n)	Percentage (%)
Educational status of anaesthetist	BSc student	81	15%
	BSc anaesthetist	201	38%
	MSc student	122	23%
	MSc anaesthetist	114	21%
	Anaesthesiologists	9	2%
	PhD anaesthetist	5	1%
Work experience of anaesthetist	< 2 year	102	19%
	2–5 year	170	32%
	> 5 year	260	49%
	Total	532	100%

### Drug related factors

During the study period, bupivacaine was used for spinal anaesthesia for all patients (Table 5). The minimum and maximum doses of bupivacaine were 7.5 mg and 20 mg respectively with the mean value of 15.41 ± 4.164 .Most patients (51%) were given the dose of bupivacaine ranging from 10 to 17.5 mg. The most frequent use of local anaesthetic drug baricity was isobaric (84%) and hyperbaric bupivacaine (16%) respectively. Most of the anaesthetists (71%) did not use adjuvants .Of 532 patients, 152 (29%) patients were given adjuvant and the most frequent adjuvants used was pethidine (*n* = 92, 61%) followed by fentanyl (*n* = 59, 39%) and morphine (*n* = 1, 1%) respectively (Table 5).

**Table 5:** Drug related factors (*N* = 532)

Variables	Category		Frequency(n)	Percentage (%)	
Type of local anesthetics(drugs)	Bupivacaine		All(532)	100%	
Dose of bupivacaine in mg	5–10 mg		138	26%	
	11–18 mg		249	47%	
	19–25 mg		145	27%	
Baricity of the LA	Hyperbaric		86	16%	
	Isobaric		446	84%	
Use of adjuvants	Yes	Pethidine	92	61%	29%
		Fentanyl	59	39%	
		Morphine	1	0.6	
		Total	152		
	No		380	71%	

### Factors related with surgery

Cesarean section was the most common type of operation (*n* = 169, 32%) followed by orthopedic surgery (*n* = 167, 31%), urologic surgery (*n* = 80, 15%), gynecologic surgery (*n* = 64,12%), general surgery (*n* = 49,9%) and vascular surgery (*n* = 3, 1%) respectively. The majority of the operations were elective surgery (*n* = 321, 60%), whereas 211 (40%) operations were emergency surgery. Additionally, two hundred eighty (53%) of the operations were done by residents and two hundred fifty two (47%) of the operations were done by senior surgeons .The shortest duration of surgery was 0.5 h and the longest duration was 4 h respectively with a mean of 1.5 ± 0.7. The majority of operations (*n* = 449, 83%) was finished within less than or equal to two hours (≤ 2 h).

### Cerebrospinal fluid characteristics and reaching at the subarachnoid space

Cerebrospinal fluid (CSF) flow was clear for the majority of patients (*n* = 467, 88%) and bloody for sixty-five patients (12%). Most of the anaesthetists (*n* = 313, 59%) checked for the negative aspiration to mix-up CSF with local anaesthetic and to fix that they are at the accurate place of subarachnoid space. 41% of the anaesthetists (*n* = 219) did not do barbotage. The majority of anaesthetist (*n* = 463, 87%) administered local anaesthetic with 1–2 attempts (Table 6).

**Table 6:** Characteristics of cerebrospinal fluid (*N* = 532)

Variables	Category	Frequency (n)	Percentage (%)
CSF flow	Clear	467	88%
	Bloody	65	12%
Barbotage	Yes	313	59%
	No	219	41%
Number of spinal attempt	1–2 times	463	87%
	3–5 times	69	13%

### Sensory and motor block assessment after spinal anesthesia

The anaesthesia provider used various assessment methods to determine the level of spinal anaesthesia for sensory and motor blockage before surgery. During the study period, anaesthetists use temperature sensation, light touch, pin prink. The majority of sensory levels (63%) were assessed using a pin prick. Some of the anaesthetist did not check the sensory level of blockage (*n* = 72, 14%).The majority of sensory block level assessments showed a loss of sensation (*n* = 341, 64%), while a few were classified as weak sensation (6%) using a modified Holmen scale. The degree of motor blockade was evaluated using the Bromage scale, where the majority of assessments (79%) showed an inability to move the feet or knees, while a small number of patients showed full flexion of the knee and feet.

### Incidence of failed spinal anaesthesia

The overall incidence of failed spinal anaesthesia was 22.4% (*n* = 119/532) at (95%, CI = 19.0-25.9) in this study. With the failure rate of among individual hospitals; at the University of Gondar comprehensive specialized hospital 23.2% (*n* = 47/203), at Tibebe Ghion comprehensive specialized hospital 22.3% (*n* = 43/193) and at Felege Hiwot comprehensive specialized hospital 21.3% (*n* = 29/136) respectively (Table 7). With regard to incidence of failed spinal anaesthesia in different operation types, majority of failed spinal anaesthesia was recorded among cesarean Sect. (27.8%), followed by general surgery (24.5%), gynecologic surgery (20.3%), orthopedic surgery (19.8%) and no failure was recorded among vascular surgery (0%). Of 119 failed spinal anaesthesia cases 65(54.6%) was partial and 54(45.4%) was complete failure. Most of failure sixty five (54.6%) was managed by repeating of spinal anaesthesia. Only for six (5%) patients was converted to general anaesthesia (Table 7).

**Table 7:** Success and failure rate of spinal anaesthesia (*N* = 532)

Variables		Category			Frequency(n)	Percentage (%)	
Was the spinal block adequate for surgery		Success			413	77.6%	
		Failed	Partial failure		65	12.2%	22.4%
			Complete failure		54	10.2%	
Name of hospitals		Succeed		Failed			
1	UoGCSH	156(76.8%)		47(23.2%)	203	38.2%	
2	TGCSH	150(77.7%)		43(22.3%)	193	36.3%	
2	FHCSH	107(78.7%)		29(21.3%)	136	25.6%	
Measurements taken to manage failed spinalanesthesia		Repeat spinal anesthesia			65	54.6%	
		Analgesia &sedation			48	40.3%	
		Convert to general anesthesia			6	5.1%	

### Factors associated with failed spinal anesthesia

The variables that had a *p* value of less than 0.2 from bivariable logistic regression include the educational status of the anaesthetist, the performance of infiltration before the attempt of lumbar puncture, the palpability of the spinous process, the urgency of the operation, the work experience of the anaesthetist, characteristics of CSF appearance, dose of local anesthetics, baricity of the local anaesthetic used, the use of adjuvants, the vertebral approach during lumbar puncture, the site of lumbar puncture, the feeling of paresthesia during lumbar puncture, immediate change of position after spinal anaesthesia, needle size, checking by negative aspiration of CSF (barbotage) and the speed of intrathecal injection.

Urgency of operation, work experience of anaesthetist, cerebrospinal fluid (CSF) flow appearance, dose of local anaesthetics (bupivacaine), baricity of local anaesthetics and use of adjuvants with local anesthetics had significant association with failed spinal anaesthesia from the multivariable logistic regression with *p* value < 0.05(Table 8).

Multivariable logistic regression analysis showed that emergency surgery done under spinal anaesthesia is seven times more likely to fail as compared to elective surgery (AOR = 7.014,CI = 4.480-12.717) .Intratecal administration of bupivacaine of ≤ 10 mg is three times more likely to fail compared with the dose of bupivacaine ≥ 17.5 mg (AOR = 3.021,95%CI = 1.262–10.232). Additionally, spinal anaesthesia administered by an anaesthesia providers who had < 2 year of work experience was three times more likely to be fail compared with spinal anaesthesia that was administered by an anaesthesia provider who had work experience of > 5 years (AOR = 3.110 ,95%CI = 1.692–5.716) and spinal anaesthesia administered by who had work experience of 2–5 years is one point two (1.2×) times more likely to fail as compared with an anaesthesia provider who had > 5 year experience (AOR = 1.176,95% CI = 1.057–2.550 ). Additionally, being bloody CSF appearance during lumbar puncture is about eight times more likely to fail as compared with clear flow of CSF (AOR = 8.488, 95% CI = 2.529–18.491). Moreover, intrathecal administration of hyperbaric local anaesthetist is three times more likely to fail compared to intrathecal administration of isobaric local anaesthetist (AOR = 3.287, 95%CI = 3.196–8.213). Furthermore, administration of intrathecal local anaesthetist without adjuvants is five times more likely to fail compared to the use of adjuvants (AOR = 5.25, CI = 2.619–14.186) (Table 8).

**Table 8:** Multivariable analysis showing factors associated with failed spinal anesthesia (*N* = 532)

variables		Spinal anesthesia		COR(95%CI)	AOR ( 95% C.I)	*P* value
		Failed *n* (%)	Success *n* (%)			
Urgency of operation	Emergency	91(43.1%)	120(56.9%)	7.935(4.94–12.75)	7.014(4.480-12.717)	0.00001
	Elective	28(8.7%)	293(91.3%)	(1)		
Dose of bupivacaine	5–10 mg	47(34.1%)	91(65.9%)	3.228(2.31–3.92)	3. 021(1.262–10.232)	0.017
	11-18 mg	50(20.1%)	199(79.9%)	1.57(1.35–1.83)	0.163(0.262–3.083)	0.671
	19–25 mg	20(13.8%)	125(86.2%)	1		
work experience of anesthetist	< 2 year	57(55.9%)	45(44.1%)	20.7(13.23–25.9)	3.110(1.692–5.716)	0.000258
	2–5 year	47(27.6%)	123(72.4%)	6.24(2.086-6.30)	1.176(1.057–2.550)	0.003
	> 5 year	15 (5.8%)	245(94.2%)	1		
CSF characteristics	Bloody	45(69.2%)	20(30.8%)	11.95(6.8-22.24)	8.488(2.529–18.491)	0.000314
	Clear	74(15.8%)	393(84.2%)	1		
Baricity of local anesthetics	Hyperbaric	61(70.9%)	25(29.1%)	16.32(3.08–25.29)	3.287 (3.196–8.213)	0.00001
	Isobaric	58(13.0%)	388(87.0%)	1		
Use of adjuvants	No	114(30.0%)	266(70.0%)	5.494(5.03–31.55)	5.250(2.619–14.186)	0.000218
	Yes	11(7.2%)	141(92.8%)	1		

## Discussion

Spinal anesthesia is a widely used technique to providing anesthesia for lowers abdominal and extremity surgical procedures and it is believed to be working and adequate for planed surgical procedures after proper administration of local anesthetics in to subarachnoid space. It considers safe and effective with a low incidence of failure & complications [14, 31]. This study showed that incidence of failed spinal anaesthesia in Amhara National Regional Sate comprehensive a specialized hospital was 22.4% (95%,CI = 19-25.9). This finding was in line with the study done in University of Gondar specialize hospital, Gondar Ethiopia among obstetric parturient the incidence of failed spinal anaesthesia was (19.5%) [23].

Our finding was high as compared with other previous studies. Most studies found that the incidence of failed spinal anaesthesia ranged from 1 to 17% [1, 14, 18, 21]. The possible reason why this high incidence occurred in our study might be due to the fact that our study was conducted in teaching hospitals and there were anaesthesia practitioners which might contribute for the failure. Of a total of 532 patients, 15.2% patients were provided anaesthesia by practitioners with the failure rate of 46.9%. This high value could be due to large sample size in the current study where the true incidence might be picked up.

This study reveals that the incidence of failed spinal anaesthesia among each comprehensive hospitals was (23.2%) at the University of Gondar specialized comprehensive hospital, (22.3%) at Tibebe Ghion specialized comprehensive hospital and (21.3%) at Felege Hiwot specialized comprehensive hospital respectively. There was no significant difference between hospitals. The possible reason might be all of hospitals has anesthesia practitioners and have comparable level of case flow and anaesthesia providers.

In this study, failed spinal anesthesia was more common in certain types of surgeries, including; cesarean Sect. (27.8%), general surgery (24.5%), gynecologic surgery (20.3%), orthopedic surgery (19.8%), and urologic surgery (17.5%). This finding in line with Turkish studies [1]. This could be due to various factors such as technical difficulty and anatomical distortion caused by the gravid uterus and labor pain in obstetric patients [32]. Pain at the fracture site in orthopedic patients making it difficult to position them optimally, and calcification of bones and ligaments in geriatric urologic and gynecologic patients has the same effect [33]. Moreover, the reason for the high failure rate in general surgery could be due to the fact that the majority of patients were emergency where lumbar puncture could be difficult as these patients would be uncooperative during positioning and lumbar puncture.

In this study, the most common failure of spinal anaesthesia was inadequate block (12.2%). This finding was in the line with a previous study [34]. The mechanism remains unclear and the appearance of CSF fluid in the needle hub does not guarantee for the success of spinal anaesthesia after the disposition of the required dose in the CSF. It believed that loss of injectate, misplace injections and ineffective drug actions could contribute for the failure of spinal anaesthesia after CSF appearance through the needle hub and successful injection of local anaesthetic drugs [35]. This study found that being emergency surgery increases the likelihoods of spinal anaesthesia failure rate by seven times compared to elective procedures (AOR = 7.01, CI = 4.5–12.7). This finding is in accordance with previous study conducted in Ethiopia by Ashagrie et al. among obstetric parturient and another study conducted in Spain [23, 36]. This could be due to those emergent patients undergoing emergency surgery may be uncooperative and difficult to position optimally for lumbar puncture. Additionally, there might be lack of assistance and availability of adjuvants and materials during emergency procedures. In emergency scenarios, patients are frequently in labor and may move while receiving an injection, which might cause the needle to shift and deposit local anaesthetics in the wrong place and surgeons may rush to operate and begin skin incisions before sufficient blockage of local anaesthetics has occurred. This finding highlights the importance of careful patient selection and preparation before performing spinal anesthesia for emergency surgeries.

In this study, intrathecal administration of bupivacaine doses of ≤ 10 mg was significantly associated with failed spinal anaesthesia. The selection of a specific dose is dependent on different factors such as the need to minimize failure of spinal anaesthesia, post spinal hypotension and early mobilization. Anaesthetists mostly use large dose for single injection to minimize the failure of spinal anaesthesia in combination with adjuvants [35]. This study found that intrathecal administration of bupivacaine doses of ≤ 10 mg is significantly associated with failed spinal anaesthesia and likelihoods of failure increased by three times compared to ≥ 17.5 mg (AOR = 3.02,CI = 1.26–10.23). Previous studies suggested that insufficient amounts of local anesthetics can cause uneven distribution and may be responsible for failed spinal anesthesia [37]. Even when placed correctly the spread of local anaesthetic solution in the intrathecal space is unpredictable and insufficient spread may have an impact on the block’s focal point. Another multicenter prospective cohort study finding is in line with our findings which was found that bupivacaine doses below 10 mg are three times more likely to fail than doses beyond 15 mg [38].

A systematic review and meta-analysis conducted in Toronto Canada found that a conventional dose of bupivacaine should be greater than 8 mg to avoid high failure rates unless other adjuvants or intrathecal catheters are used [39] and this study reports that intrathecal administration of low dose local anesthetics plays a great role for spinal anaesthesia failure. This systematic review and meta-analysis study also found that low dose bupivacaine increase the need for analgesic supplementation during surgery [39]. Specially during cesarean section the intended dermatomal level is around T6 so there might be feeling of pain during fundal manipulations while extraction of fetus and this increases analgesia and sedation requirements. This finding emphasizes the importance of accurately dosing intrathecal bupivacaine to ensure successful spinal anaesthesia.

This study found that work experience level of the anaesthesia providers is a significant factor in the failure of spinal anesthesia. Those who administered spinal anaesthesia with less than 2 years of work experience had 3 times a higher likelihood of failure compared to those with more than 5 years of experience (AOR = 3.1,CI = 1.7**–**5.7). This finding is consistent with previous studies conducted in a Nigerian teaching hospital shows that the failure of spinal anaesthesia was highly associated with work experience of the anaesthetist [17]. Moreover, short years of work experience could cause incorrect patient positioning, inappropriate needle insertion, inappropriate dose, loss of injectate and misplace injection [40],

Additionally, this study found that anaesthesia providers who have work experience of two to five years had got likelihood of failure rate 1.2 times as compared to those with have greater than five years of work experiences (AOR = 1.2,CI = 1.1**–**2.6). Ashagrie et al. also reports that failure of spinal anesthesia has significant association with work experience of anaesthetist [23]. The possible reasons might be that experience is the way of learning and experienced anaesthetist can use the appropriate dose of drugs for respective patients and type of surgery but less experienced may not do this. In this regard, experience play a major role in all aspect of the procedure including, drug selection, optimum positioning of patients during and after spinal anaesthesia procedure. This finding highlights the importance of adequate training and experience sharing for anaesthesia providers who perform spinal anaesthesia since selection of the proper dose of local anesthetic agent for a given procedure still remains an art.

This study found that an appearance of bloody mixed CSF flow during lumbar puncture increases the probability of failure by eight times as compared with clear CSF flow (AOR = 8.5, CI = 2.5–18.5). Our finding was in line with previous studies [23, 41]. Alabi et al. reported that bloody CSF indicates inaccurate placement of spinal needle into blood vessel would significantly contribute for the likelihood of block failure [41]. The study done in South Africa found that the presence of bloody cerebrospinal fluid (CSF) during the initial attempt of spinal anesthesia was strongly linked to failure the block [15]. Another study done in North Carolina was found that intrathecal administration of local anaesthetics in the absence of free flow CSF and in the appearance of blood ticked CSF the failure rate was high as compared with clear flow of CSF [24]. This has important implications for both clinical practice and research, as it increases the risk of incorrect placement of the needle into a blood vessel and subsequent complications from the injection of bupivacaine. So the clinicians give emphasize that careful observation and interpretation of CSF appearance during lumbar puncture is essential to improve the success rate of spinal anesthesia.

Additionally, this study found that using hyperbaric local anaesthetics for intrathecal injection has significant association for failed spinal anesthesia by three times as compared to isobaric local anesthetics (AOR = 3.3,CI = 3.2**–**8.2). Our result supported by McClure et al. since they reported that as a solution with a density within the normal range of that of CSF (‘isobaric’) will virtually guarantee block of the lower limbs surgery [42]. Similar prospective comparative study was comparing between hyperbaric and isobaric local anaesthetics revealed that failure rate was seen in higher among hyperbaric solutions groups [22].

Solutions with a density greater than that of CSF (hyperbaric) move very definitively under the combined influence of gravity and the curves of the vertebral canal. Sukhani et al. found that there was not failure among isobaric LA as compared with hyperbaric [11, 43]. Possible reason may be most of patients were changing position to different sides after spinal anesthesia administered, the solution will spread ‘down’ the slope under the effect of gravity to pool at the ‘lowest’ point of the thoracic nerves. The practical significance of this finding is that anaesthetist needs to be cautious when selecting the local anesthetic solution for spinal anesthesia and should consider the patient’s posture during the operation and the desired level of anesthesia. Isobaric local anesthetics may be more suitable for lower limb surgery since they have a density comparable to that of cerebrospinal fluid and less affected by positioning and gravity.

This study also found that using adjuvants such as pethidine, fentanyl, and morphine with local anesthetics for intrathecal injections can significantly reduce the failure rate of spinal anesthesia. Conversely, not using adjuvants can increase the failure rate by five times (AOR = 5.25, CI = 2.62**–**14.2). Fuzier et al. also reported in their study that the absence of adjuvant medication with the local anesthetics is a significant predictor factor for failed spinal anesthesia [21].

A multicenter prospective study done in France; by Fuzier et al. founds that the absence of adjuvant medication with the local anesthetic increase the failure rate of spinal anaesthesia by two times [44]. The use of adjuvants can help patients feel more comfortable and improve the success of the procedure for anesthesia providers. It has been reported that adjuvants potentiate local anaesthetic drugs and decrease the requirement for additional analgesia during surgery [40]. This finding highlights the importance of selecting the appropriate type of local anesthetic and using adjuvants to enhance adequate blockage of spinal anesthesia.

In this study, we did not find association between failed spinal anesthesia and socio demographic variables like age ,ASA statues and BMI ;Even though the study done in South Africa among obstetric mothers found that there were significant relation between failed spinal anesthesia and BMI [15]. One study reports that failed spinal anaesthesia influence by obesity independently is still controversial [45]. Ashagrie et al. reports on their study among obstetric mothers they did not find significant association between socio demographic variables and failed spinal anaesthesia like ASA status, BMI [23]. The possible reason why obesity was not significantly associate with failed spinal anesthesia in case of our study might be there were small number of overweight patients 19 (3.6%) were BMI > 30 kg/m2 .

In this study, we did not find significant association between failure of spinal anaesthesia and sit of lumbar puncture. Even though some studies reports that like Nigerian teaching hospital use of the L4/L5 interspace for lumbar puncture site increase chance of spinal anesthesia failure rate by two times [17]. However, a study done in Chicago supports our result since they did not found that significant difference in failure rates between spinal anaesthesia administered in the lateral and sitting position as well as site of lumbar puncture [1, 22]. The possible reason might be here in our study majority of patients were done with isobaric local anesthetic which is not affected by site and position.

In this study, it is good that the conversion of failed spinal anaesthesia to general anaesthesia was 5% and it is low as compared with other studies .One study reports that 80% of completely failed spinal anesthesia was converted into general anesthesia [31] and the Royal College of anesthetists recommended, in possession of best practice, that the changing rate from spinal anesthesia to GA should be ˂3% since conversion to GA increases the morbidity and mortality. Deshpande and Idriz ,Fettes, Jansson et al. were found that repeating spinal anesthesia after unsuccessful full intrathecal anaesthesia is a good way and safe alternatively adjuncts with analgesia and light sedation than general anaesthesia since general anesthesia has cardiopulmonary compromization and related airway complications [14, 46].

### Limitation of the study

The anaesthetists who managed the patients were not blinded during data collection.

### Strength of the study

This is a multicenter study and large sample size of the study. Additionally, unlike the previous studies, this study included varies surgical specialties and assessed additional factors such as vertebral needle approach (midline, paramedian), speed of injection, CSF flow (flow seen, no flow), barbotage, time taken to achieve complete block, block status (partial, complete), management of failed spinal anaesthesia (spinal anaesthesia repeated, converted to GA).

### Conclusion and recommendation

The overall incidence of failed spinal anesthesia was high in Amhara National Regional State Comprehensive Specialized hospitals. Emergency surgery, dose of bupivacaine of < 10 mg, work experience of anaesthetist < 2 year, being bloody CSF appearance during lumbar puncture, intrathecal administration of hyperbaric local anaesthetist and intrathecal local anesthetics without adjuvants had strong association with failed spinal anaesthesia. It is recommended that emphasis should be given for anaesthesia trainees on spinal anaesthesia. Additionally, anaesthesia providers should use appropriate dose of local anaesthetics and use adjuvants. Moreover, simulation training should be given on spinal anaesthesia for all anaesthesia trainees.

## Data Availability

All the raw data for this paper are available upon reasonable request to the corresponding author.

## Abbreviations

AOR: Adjusted odds ratio
ASA: American society of anaesthesiology
BMI: Body mass index
COR: Crude odds ratio
CSF: Cerebrospinal fluid
FHCSH: Felege hiwot comprehensive specialized hospital
FSA: Failed spinal anaesthesia
GA: General anaesthesia
LA: Local anaesthesia
SA: Spinal anaesthesia
TGCSH: Tibebe ghion comprehensive specialized hospital
UoGCSH: University of gondar comprehensive specialized hospital
